# The role of gut microbiota in chronic intestinal pseudo-obstruction: exploring fecal microbiota transplantation as a treatment option

**DOI:** 10.1080/19490976.2025.2610597

**Published:** 2026-01-07

**Authors:** Giada De Palma, Anna Costanzini, Vidhyalakshmi Mohan, Sacha Sidani, Zarwa Saqib, Marc Pigrau, Jun Lu, Natalia Causada Calo, Ines Pinto-Sanchez, Elena F. Verdu, Margaret Marcon, Giovanni Barbara, Vincenzo Stanghellini, Roberto De Giorgio, Stephen M. Collins, Premysl Bercik

**Affiliations:** a Farncombe Family Digestive Health Research Institute, Department of Medicine, McMaster University, Hamilton, Canada; b Department of Translational Medicine, University of Ferrara, Ferrara, Italy; c Division of Gastroenterology, The Hospital for Sick Children, University of Toronto, Toronto, Canada; d IRCCS University Hospital, Bologna, Italy; Department of Medical and Surgical Science, University of Bologna, Bologna, Italy

**Keywords:** Chronic intestinal pseudo-obstruction, severe constipation, microbiome, fecal microbiota transplantation

## Abstract

Chronic intestinal pseudo-obstruction (CIPO) is characterized by bowel dilation and obstructive symptoms without any structural blockage. Although the microbiota is known to affect gastrointestinal function, its role in CIPO is poorly understood. We aimed to characterize the CIPO microbiota, investigate its role in disease expression and explore the therapeutic role of fecal microbiota transplantation (FMT). CIPO patients (*n* = 14) and healthy controls (HC, *n* = 12) were recruited from Italy and Canada. Microbiota profiles and functions were assessed by 16S rRNA sequencing and PICRUSt. Germ-free NIH Swiss mice were colonized with HC and CIPO microbiota, their intestinal transit and bowel distension were assessed by videofluoroscopy and computed tomography (CT), and the expression of host genes by NanoString®. The CIPO microbiota exhibited reduced microbial diversity with dominance of Proteobacteria and altered metabolic function. Mice with CIPO microbiota developed marked bowel distension and slow intestinal transit associated with altered expression of multiple genes related to immunity, the intestinal barrier and neuromuscular function. FMT from a HC improved the microbiota profile, intestinal transit and bowel distension in both CIPO mice and a selected CIPO patient, in whom a marked clinical improvement was sustained for 8 y. Thus, our findings support the use of microbiota-directed therapies to induce clinical improvement in CIPO patients.

## Introduction

Chronic intestinal pseudo-obstruction (CIPO) is a rare and debilitating condition characterized by obstructive symptoms of bloating, pain, distension, and constipation that occur in the absence of structural blockage.^1^ Idiopathic CIPO arises as a result of a primary impairment of the neurogenic or myogenic mechanisms that control intestinal motility. Its morbidity is related to slow intestinal transit and impaired nutrition, that often requires enteric or parenteral support.^2^ Although widely used, prokinetic drugs seldom provide sufficient clinical relief under these conditions.

The intestinal microbiota is an important factor in the regulation of gastrointestinal (GI) motility, and its disruption has been implicated in functional GI motility disorders in adults and children.^3^ There is a bidirectional relationship between microbiota composition or metabolic activity, and intestinal transit. Slow transit alters bacterial composition and short-chain fatty acid production in the distal colon in humans^4^ and in an *in vitro* system.^5^ Antibiotic administration often induces diarrhea in patients,^6^^,^^7^ although microbiota depletion has been shown to delay intestinal transit in animal models.^8^^,^^9^ Thus, the primary alteration in motility in CIPO is likely to change the microbiota profiles that, in turn, further influence intestinal motility. A recent study demonstrated increased breath hydrogen excretion in CIPO patients^10^ implicating methane as a mediator of slow intestinal transit.^11^ In addition, another study in pediatric CIPO patients exhibited alterations in the colonic mucosa-associated microbiota and in the expression of serotonin-related genes.^12^ Similarly, one pilot study in adult CIPO patients demonstrated improved tolerance of naso-jejunal feeding and modest improvements in pain and bloating following fecal microbiota transplantation (FMT).^13^ Thus, although the putative role of the microbiota in CIPO has been acknowledged, this remains an understudied field.^14^ Moreover, the microbiome of adult CIPO patients has not been profiled, and the functional relevance of the microbiome in the expression of CIPO has not been established.

The objectives of the present study were three-fold (Supplementary Figure 1). First, we examined the composition of the microbiota in CIPO patients and age- and sex-matched healthy controls (HC), recruited from two centers in Canada and Italy. Patients were analyzed separately and collectively, considering the presence or absence of colectomy, a common procedure in CIPO patients. Second, in a proof-of-concept study, we examined the functional impact of the microbiota from a single CIPO patient and a healthy control using a humanized mouse model. Third, our findings prompted us to investigate the efficacy of fecal microbiota transplantation (FMT) in the management of this patient. Our results demonstrate dysbiosis in an international cohort of CIPO patients, provide evidence of a functional role of the microbiota in this disorder, and suggest that the microbiota is an appropriate target for therapies, that include FMT.

**Figure 1.**
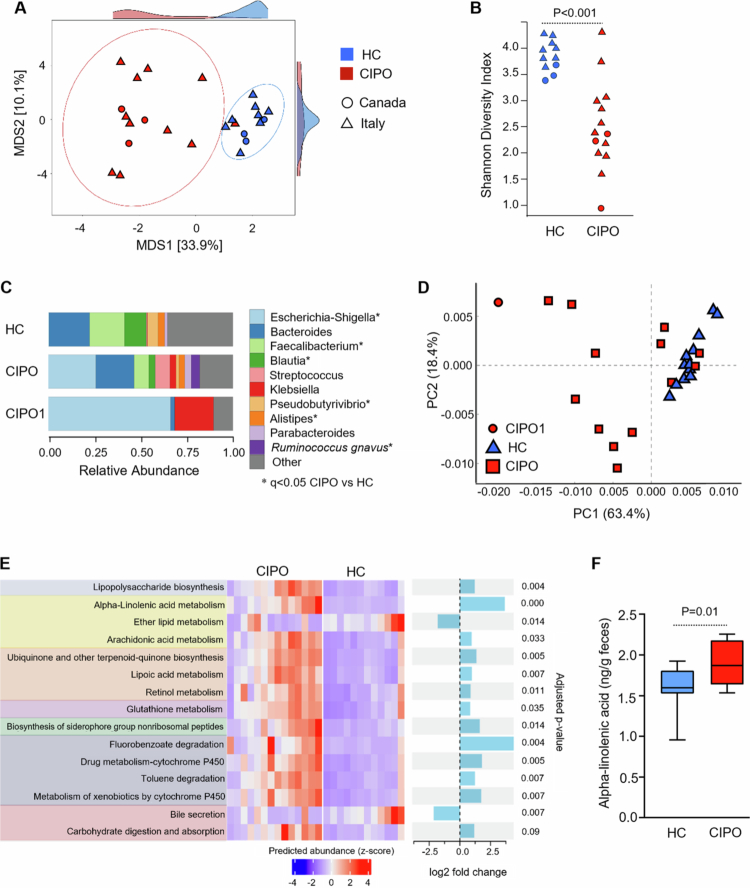
Microbiome profiles and function are altered in patients with CIPO. (A) Multidimensional scaling (MDS) plot of the Atchison distance metric constructed between HC (*n* = 12) and CIPO patients (*n* = 14) from both Canada and Italy. The type of microbiota (HC, CIPO) drove the clustering (*P* = 0.001, PERMANOVA). The side panel density plots show the distribution of samples along each axis of the ordination plot. (B) Alpha diversity, measured as the Shannon diversity index, in HC and CIPO patients from Italy and Canada. *P*-values were calculated using Mann–Whitney U test. (C) Taxonomic composition at the genus level of the microbiome of HC, CIPO patients and the index patient (CIPO1). The data are shown as relative abundances. *P*-values were calculated with a multivariate regression model using MaAsLin2^15^, and a *P*-value <  0.05 after false discovery rate (FDR) correction using Benjamini–Hochberg procedure was considered significant. (D) Principal component analysis (PCA) plot comparing the pathway-level profiles of HC and CIPO patients predicted using PICRUSt2.^16^ The type of microbiota (HC, CIPO) drove the clustering (*P* = 0.001, ANOVA). The graph was produced with STAMP (v 2.1.3).^17^ Red circles indicate three independent samples of the index CIPO patient. (E) Microbial metabolic pathways that differed between HC and CIPO patients. The functional potential of HC and CIPO bacterial communities was predicted on the basis of marker gene sequencing profiles with PICRUSt2.^16^ Differential abundance analysis was performed using LinDA^18^ within ggpicrust2.^19^ The heatmap displays individual data for all differentially abundant pathways. The color intensity represents z-scores, calculated from the relative abundance of each pathway. *P* values were adjusted for FDR using Benjamini–Hochberg procedure, and a *P*-value < 0.05 after correction was considered significant. (F) Alpha-linolenic acid was assessed in stool samples of HC and CIPO patients. The data were analyzed with an unpaired Mann‒Whitney U test. A *P*-value < 0.05 was considered statistically significant.

## Materials and methods

### Sex as a biological variable

Our study included both female and male patients at an approximately 50% ratio; however, this study is not adequately powered to look into the effect of sex as a variable. Similarly, we examined both male and female animals, with similar results observed in both sexes.

### CIPO patients and healthy controls

The Canadian CIPO cohort consisted of 3 patients, and the Italian CIPO cohort consisted of 11 CIPO patients (for demographic and clinical details, see Table 1). Most CIPO patients were on modified or liquid diets, some were on total parenteral nutrition (TPN) (Table 1).

**Table 1.** Clinical characteristics of CIPO patients.

Patient	Country	Demographics/Clinical history	Surgeries
CA1	Canada	Male; 21 y. Age at diagnosis: 2; abdominal distension and pain; recurrent occlusive epizodes	Total colectomy, ileorectal anastomosis + adhesiolysis
CA2	Canada	Female; 45 y. Age at diagnosis 37, severe constipation, abdominal pain and bloating	Sigmoid colon resection, hysterectomy, adhesiolysis
CA3	Canada	Male; 39 y. Age at diagnosis 14, abdominal pain, constipation, recurrent episodes	Appendectomy, cholecystectomy
ITA1	Italy	Male; 68 y. Age at diagnosis: 28; abdominal distension and pain; recurrent sub-occlusive episodes; intrinsic neuropathy; esophageal hypocontractility; oral feeding (modified)	Total colectomy + resection of duodeno-jejunal segment
ITA2	Italy	Female; 59 y. Age at diagnosis:15; abdominal distension and pain; recurrent sub-occlusive episodes; intrinsic neuropathy; esophageal hypocontractility; oral feeding (modified)	Total colectomy + permanent (decompressive) ileostomy; adhesiolysis
ITA3	Italy	Male; 29 y. Age at diagnosis: 6; abdominal distension and pain; intrinsic neuropathy on manometry; esophageal involvement; TPN	Total colectomy + Roux-en-Y gastro-entero-anastomosis
ITA4	Italy	Male; 30 y. Age at diagnosis: 8; abdominal distension and pain; no phase II + post-prandial hypomotility on manometry, esophageal involvement: GERD; oral feeding (modified)	Pyloric stenosis; then, ileal volvolus derotation; enteroplication (Noble procedure)
ITA5	Italy	Female; 42 y. Age at diagnosis: 31; abdominal distension and pain; Intrinsic neuropathy (manometry); esophageal involvement; oral feeding (modified)	Total colectomy + temporary ileostomy, then ileo-rectal anastomosis
ITA6	Italy	Female; 69 y. Age at diagnosis: 40; abd distension and pain: intrinsic neuropathy on manometry; esophageal involvement; oral feeding (modified); i.v. rehydratation therapy	Appendectomy, duodenum-jejunal resection + cholecystectomy
ITA7	Italy	Male; 60 y. Age at diagnosis: 42; abdominal distension and pain; intrinsic neuropathy on manometry; PN 4/week + modified oral intake	Subtotal colectomy + adhesiolysis
ITA8	Italy	Male; 43 y. Age at diagnosis: 8; abdominal distension and pain; chronic sub-occlusive status; intrinsic neuropathy on manometry; esophageal involvement; PN + oral feeding (modified)	Appendectomy; volvulus derotation, adhesiolysis, gastro-jejunostomy
ITA9	Italy	Female; 54 y. Age at diagnosis: 5; abdominal distension and pain; intrinsic neuropathy on manometry; oral feeding (modified)	Total colectomy with ileo-rectum anastomosis; rectal prolapse repair
ITA10	Italy	Female; 74 y. Age at diagnosis: 52; abdominal distension and pain: intrinsic neuropathy on manometry; esophageal involvement; oral feeding (modified)	Appendectomy, adhesiolysis, right colectomy with latero-lateral ileo-left colostomy
ITA11	Italy	Female; 26 y. Age at diagnosis:16; abdominal distension and severe pain; chronic sub-occlusive status; intrinsic neuropathy; esophageal involvement; TPN	Subtotal colectomy, adhesiolysis, permanent ileostomy
ITA12	Italy	Male; 35 y. Age at diagnosis:18; abdominal distension and pain, chronic sub-occlusive status; intrinsic neuropathy; esophageal involvement; TPN	Jejunostomy
ITA13	Italy	Female; 20 y. Age at diagnosis: 17; severe constipation; abdominal distension and severe pain; recurrent sub-occlusive episodes; colon biopsies: massive mast cell infiltrate; modified oral diet	Laparoscopic derotation of the left ovary funiculus due to a benign cystic lesion

The Canadian patients were recruited from McMaster University Medical Center Digestive Diseases Clinic, while the Italian patients were recruited from the Laboratory of Functional Gastrointestinal Disorders of St. Orsola Malpighi Hospital, Bologna, or the Division of Internal Medicine, St. Anna Hospital, University of Ferrara.

As controls, we recruited 3 Canadian and 9 Italian healthy volunteers, who were age- and sex-matched. Healthy volunteers had no history of organic disease, immune deficiency, major abdominal surgery, use of immunosuppressants, glucocorticoids or opioids.

### Study approvals

The studies were approved by the respective ethics board of the three institutions. All patients and healthy volunteers provided written informed consent. In addition, the index patient provided separate written consent for medical photographs to be taken and used for publication.

### Healthy stool donor

The healthy donor for fecal microbiota transplantation (FMT) was recruited from the St. Joseph’s Healthcare centre in Hamilton, Ontario, Canada, and was selected from the bank of available healthy donors who previously participated in other clinical trials of FMT.^20^

### Microbiota analysis

Human stool samples were collected and immediately frozen at −80 °C. Murine fecal samples were collected 3 weeks after colonization and immediately frozen at −80 °C. In addition, the contents of the colon and cecum were collected at sacrifice. Total genomic DNA was extracted from the stool samples as previously described.^21^ Amplification of the V3 region of the 16S rRNA gene, and Illumina sequencing were performed as previously described.^21^^,^^22^ The data were analyzed following the pipelines of the dada2^23^ and Phyloseq packages (1.28)^24^ for R (3.6.1). Taxonomic assignments were performed using the RDP classifier^25^ with the Silva small subunit Ref. NR99 138.1 database^26^^,^^27^ (2020) training set. Samples with <1000 reads were removed from the analysis, resulting in an average reads/per sample of 99925. Metagenomic functional content was predicted from 16S rRNA gene profile of samples using PICRUSt 2.0 (v. 2.4.1)^16^ and visualized using ggpicrust2 package for R.^19^ All the results were corrected for multiple comparisons, allowing 5% of False Discovery Rate.

### FMT protocol

Stool from the healthy donor was prepared for FMT as previously described^20^ and stored at −80 °C. FMT was administered via the jejunostomy tube four time daily for two weeks (50 cc daily), then every other day for two weeks (50 cc Q2 days), and subsequently once weekly for 2 months (50 cc Q week) and then once monthly for 3 months. After initial supervised administration by a nurse practitioner at the clinic, subsequent doses were auto-administered by the patient at home.

### Clinical assessment of the index CIPO patient

Clinical assessment was performed at baseline (7 d before initiating FMT) and weekly for 4 weeks and bi-monthly subsequently. Transumbilical abdominal girth was measured 7 d before and 3 weeks after FMT. Objective and validated self-administered questionnaires were completed before and after FMT. The RAND-36 Health Survey, a validated and widely used tool to measure health-related quality of life, was administered 7 d before, 3 and 20 weeks after initiating FMT.^28^ The Hospital Anxiety and Depression Scale, a validated tool to measure the contribution of co-morbid anxiety and depression to the experience of suffering in the medical setting,^29^ as well as the Bristol Stool Form Scale to assess stool consistency,^30^ were completed 7 d before and 20 weeks after FMT. Finally, the average number of stools per day during the last week was also recorded 7 d before and 20 weeks after FMT. Adverse events were assessed and recorded at each visit. The patient was followed for 8 y.

**Table 2.** Taxa (at genus level) that differed between CIPO patients and healthy controls.

Feature	Value	Coef	Stderr	Qval
Lachnospiraceae.UCG.003	CIPO	−2.55	0.21714	5.33E-10
Erysipelotrichaceae.UCG.003	CIPO	−2.88	0.312922	4.61E-08
Monoglobus	CIPO	−3.25	0.411511	5.98E-07
Christensenellaceae.R.7.group	CIPO	−6.00	0.7811	7.59E-07
X.Eubacterium.ventriosum.group	CIPO	−3.41	0.450226	7.92E-07
Family.XIII.AD3011.group	CIPO	−2.69	0.361771	9.05E-07
Escherichia.Shigella	CIPO	8.11	1.187897	3.42E-06
Lachnoclostridium	CIPO	−2.62	0.38862	3.83E-06
Dorea	CIPO	−6.09	0.932243	5.59E-06
Subdoligranulum	CIPO	−7.15	1.185253	1.79E-05
Fusicatenibacter	CIPO	−5.01	0.83506	1.79E-05
Lachnospiraceae.ND3007.group	CIPO	−3.18	0.5383	2.06E-05
Family.XIII.UCG.001	CIPO	−2.02	0.363819	4.77E-05
X.Eubacterium.hallii.group	CIPO	−5.95	1.103106	6.74E-05
Anaerostipes	CIPO	−4.64	0.908073	0.000133
Lachnospira	CIPO	−4.67	0.953278	0.000221
Lachnospiraceae.UCG.010	CIPO	−3.60	0.759506	0.000317
Faecalibacterium	CIPO	−7.59	1.634524	0.000365
Butyricicoccus	CIPO	−1.77	0.379193	0.000365
Phascolarctobacterium	CIPO	−2.53	0.570716	0.000595
Pseudobutyrivibrio	CIPO	−5.47	1.294163	0.000976
UCG.005	CIPO	−4.08	0.973752	0.001026
UCG.002	CIPO	−3.27	0.818836	0.00165
Blautia	CIPO	−4.57	1.193146	0.00241
Alistipes	CIPO	−6.15	1.617154	0.002494
X.Eubacterium.ruminantium.group	CIPO	−3.12	0.84862	0.003295
Collinsella	CIPO	−4.43	1.240165	0.004152

### Humanized mouse model

Germ-free NIH Swiss mice (*n* = 30), 8–10 weeks old, of both sexes (provided by the Axenic Gnotobiotic Unit of McMaster University) were colonized by oral gavage using diluted human fecal samples (1:10 in sterile PBS, 200 µL per mouse) obtained either from the CIPO patient (*n* = 12) or the healthy control (*n* = 8). The mice were kept on a 12:12 h light‒dark cycle with free access to food and water. Three weeks later, gastrointestinal transit and abdominal CT scan experiments were performed 48 h apart. After this initial assessment, ten mice colonized with CIPO microbiota were allocated for FMT experiments and received either autologous FMT (*n* = 5) or FMT from mice colonized with healthy donor microbiota (*n* = 5). The FMT was administered orally three times a week for 2 weeks, using diluted cecum contents from donor mice. Gastrointestinal transit and abdominal CT scan experiments were then performed again. Two mice were lost during the experiments due to fight wounds, leaving 4 mice per group. The mice were sacrificed after the functional tests, and samples were collected for further analysis. The number of animals per group was determined based on power calculations using data on gastrointestinal motility from our previously published studies, including ours.^31‐33^ We assumed an effect size of 1.45 and a standard deviation of 3.04, with *α* = 0.05 and a target power of 80%, which resulted in a sample size of 8 animals per group. For the FMT animal studies, we aimed to minimize the number of animals used, assessing each mouse before and after receiving either a heterologous or autologous FMT.

All the experiments were approved by the McMaster University Animal Research Ethics Board.

### In vivo gastrointestinal transit measurement

Gastrointestinal transit was measured *in vivo* using the previously described videofluoroscopic bead method.^33^ Recorded images were stored on a digital recorder (DSR-25, Sony of Canada Ltd., Toronto, ON, Canada) and analyzed using ImageJ software (U.S. National Institutes of Health, Bethesda, Maryland, USA).^34^ The location of each bead within a given animal was determined. Each bead was given a score depending on its location within the gastrointestinal tract as previously described.^33^ The investigator was blinded to the colonization status and microbiota profile of each mouse.

### Oral contrast-enhanced abdominal CT scan

Abdominal CT scans were performed as previously described.^35^ Briefly, the mice underwent an abdominal CT scan 48 hours after the videofluoroscopic transit measurement. The mice were orally gavaged with 0.2 mL of Diatrizoate Meglumine and Diatrizoate Sodium solution USP (Gastrografin, E-Z EM Canada, Montreal, QC, Canada) 2 h prior to image acquisition. CT images were acquired on an X-SPECT (Gamma Medica, Northridge, CA, USA) at the Centre for Pre-Clinical and Translational Imaging at McMaster University (Hamilton, ON, Canada). Axial, coronal and sagittal images of the thoraco-abdominal region were generated and processed using Osirix Imaging Software for iOS version 7.5 (Pixmeo SARL, Bernex, Switzerland). The maximal diameter of the small bowel loops (a measure of intestinal distension) was determined by simultaneously examining the axial, sagittal and coronal planes of the abdomen using the 3D MPR feature. The cecum and stomach were highlighted using the polygon tool in 15–30 axial cuts that were evenly distributed orally and caudally, and the volume was calculated using the pre-programmed software volume measurement tool.

### Alpha-linolenic acid assessments

Alpha-linolenic acid was measured in the stool and cecal samples by ELISA after a 1:10 (w:vol) dilution in PBS following the manufacturer’s instructions (MyBiosource, Inc., San Diego, CA, USA).

## Results

### **Microbiome profiles in CIPO patients and healthy controls**

Microbial profiles differed between the Canadian and Italian CIPO patients compared to controls (Figure 1A), with a lower Shannon diversity score in the patients (Figure 1B). There was no significant difference between the two patient cohorts, thus we merged the data for this and all subsequent analyses. CIPO patients’ microbiome was dominated by members of the Proteobacteria (renamed Pseudomonadota) phylum such as *Escherichia-Shigella* spp (Q = 3.42*10^−6^) and had a lower relative abundance of many members of the Firmicutes phylum including *Faecalibacterium* spp, *Dorea* spp, *Blautia* spp., and *Pseudobutyrivibrio* spp. (Figure 1C, Table 2). We found that patients (*n *= 9) who underwent partial (*n *= 3) or total colectomy (*n *= 6) had a similar microbiome composition compared to patients without colectomy (*n* = 5; Table 1, and Supplementary Figure 2), albeit with a decrease in the relative abundance of several strict anaerobes, most belonging to the Firmicutes phylum, such as *Oscillospira*, *Flavonifractor*, *Anaerotruncus*, and *Colidextribacter* spp. among others, as well as *Alistipes* spp. (Supplementary Table 1).

**Table 3.** Digestive and extra-digestive symptoms in the index CIPO patient.

Questionnaire item	Before FMT	Week 3	Week 20
**RAND-36 Health Survey scores (points)**			
1. Physical functioning	10	20	75
2. Role limitations due to physical health	0	0	0
3. Role limitations due to emotional problems	0	0	0
4. Energy/fatigue	15	15	30
5. Emotional well-being	32	44	40
6. Social functioning	0	25	50
7. Pain	0	10	55
8. General health	25	15	25
**Hospital anxiety and depression**			
Anxiety score (points)	14	–	13
Depression score (points)	10	–	8
**Stool parameters**			
Bristol Stool Form Scale	6	–	6
Stool frequency (bowel movements per day)	2	–	5

**Figure 2.**
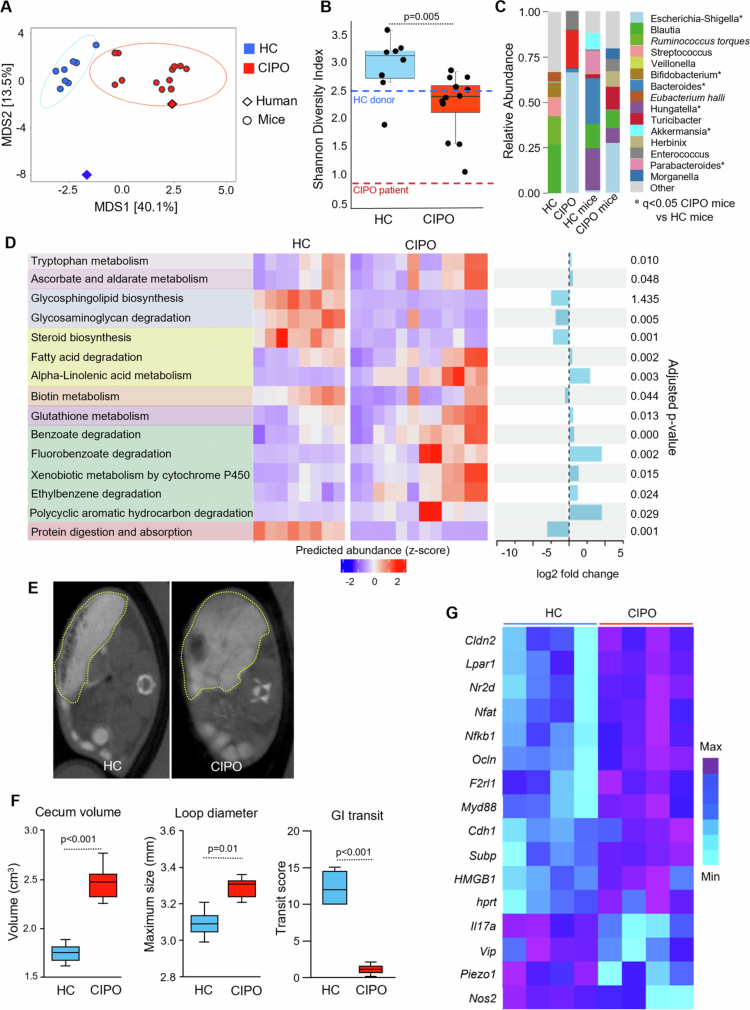
Altered microbiome profiles in CIPO mice are associated with bowel distension and slow transit. (A) Multidimensional scaling (MDS) of the Atchison distance metric constructed between mice colonized with the fecal microbiota of an HC (*n* = 8) and the index CIPO patient (*n* = 12) (CIPO). The type of microbiota (HC, CIPO) drove the clustering (*P* = 0.001, PERMANOVA). (B) Alpha diversity, measured as the Shannon diversity index, in HC and CIPO mice. *P-*values were calculated using Mann–Whitney U test. (C) Taxonomic composition at the genus level in HC and CIPO mice. The data are shown as relative abundance. *marks significant differences between HC and CIPO mice. *P*-values were calculated with a multivariate regression model using MaAsLin2 ^15^, and a *P*-value < 0.05 after false discovery rate (FDR) correction using Benjamini–Hochberg procedure was considered significant. (D) Microbial metabolic pathways that differed between HC and CIPO mice. The functional potential of bacterial communities was predicted on the basis of marker gene sequencing profiles with PICRUSt2. Differential abundance analysis was performed using LinDA^18^ within ggpicrust2.^19^ The heatmap displays individual data for all differentially abundant pathways. The color intensity represents z-scores, calculated from the relative abundance of each pathway. *P*-values were adjusted for FDR using Benjamini–Hochberg procedure, and a *P*-value < 0.05 after correction was considered significant. (E) Representative CT scan images of HC and CIPO mice. The dotted line outlines the mouse cecum. (F) Cecum volume, small intestine loop diameter and GI transit scores of HC and CIPO mice. The data were analyzed with an unpaired Mann‒Whitney U test. A *P*-value < 0.05 was considered statistically significant. (G) Heat map of genes differentially expressed in the colons of HC (*n* = 4) and CIPO (*n* = 4) mice. Only genes that were significantly different between CIPO and HC mice are shown. A *P*-value < 0.05 was considered statistically significant.

The inferred functional profile of the patients’ microbial communities (Figure 1D,E) showed enrichment in genes associated with oxidative stress (including metabolism of glutathione, lipoic acid, retinol, arachidonic acid, and cytochrome P450 activity), alpha‒linolenic acid metabolism, fluorobenzoate degradation, and carbohydrate degradation. Similarly, pathways related to the bloom of *Escherichia–Shigella* species (such as lipopolysaccharides biosynthesis) were enriched in CIPO patients (Figure 1E). Stool sample analyses confirmed that alpha-linolenic acid was higher in CIPO patients than in healthy controls (Figure 1F). When we specifically investigated methane metabolism pathways (KEGG Ortholog 0068), we found that the CIPO microbiome had a lower relative abundance of three related enzymes: heterodisulfide reductase (EC1.8.98.1), trymethilamine methyltransferase (EC 2.1.1.250), and CO dehydrogenase (EC 1.2.7.4). However, these alterations might not directly reflect a decrease in methane production but rather a modification to dietary fiber content that would impact bacterial fermenters, given that these patients suffer from severe bloating.

### Index case for proof-of-concept study

A male patient of Caribbean descent, suffering from constipation since the age of 9 months, was diagnosed clinically and histologically with myogenic CIPO (CIPO-1/CA1 in Table 1) at age of 2 y at The Hospital for Sick Children in Toronto. Extensive investigation revealed repeated radiographic evidence of intestinal obstruction without a specific transition point, characteristics of CIPO, delayed colonic transit and slow gastric emptying. Multiple treatment modalities were attempted over the course of his illness, including antiemetics and prokinetics, without significantly improving his condition. Additionally, enteral nutritional support was given orally at different time points during childhood and adolescence. He had extensive surgical history which started at the age of 10 y, with laparotomy and sigmoid colostomy. At age 15, he had closure of the initial colostomy, transverse and ascending colostomy, followed by partial descending colon resection and closure of the colostomy one year later. Histopathologic examination of the removed colon showed thinning of the circular muscle layer, compatible with myopathic CIPO. The same year, he had two laparotomies with lysis of the adhesions. He underwent a further laparotomy at age 17 with lysis of adhesions and ileotomy, which was reversed the following year because of stomal prolapse. Subsequently, the patient underwent colectomy with ileorectal anastomosis. A gastrostomy venting tube and jejunal feeding tube were provided before his care was transferred to the adult gastroenterology service at McMaster University Medical Center.

During his initial visit (September 2014), he exhibited malnutrition and severe abdominal distension and complained about severe bloating, abdominal pain and discomfort, despite passing several liquids to semiliquid stools daily. Prucalopride provided minimal symptomatic improvement. A glucose hydrogen breath test was positive for small intestinal bacterial overgrowth (SIBO). Antibiotic treatment with tetracycline resulted in transient symptom improvement with a decrease in abdominal girth, repeated treatments with tetracycline and other antibiotics were not successful.

**Figure 3.**
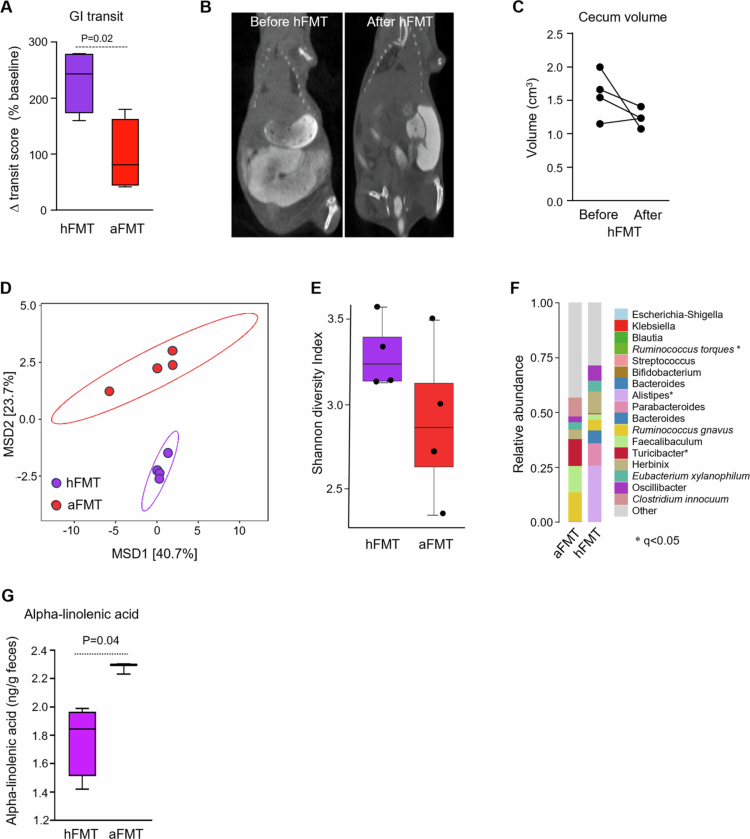
FMT with HC microbiota improves CIPO-like phenotype in humanized mice. GI transit score change after autologous FMT (aFMT) or heterologous FMT (hFMT) in mice colonized with CIPO microbiota. The values were calculated as a percentage of the baseline transit score. The data were analyzed with an unpaired t-test. A *P*-value < 0.05 was considered statistically significant. (A) Representative CT scan images of a CIPO mouse before and after hFMT. (B) Cecum volume of CIPO mice before and after hFMT. The data were analyzed with paired t-test. (C) Multidimensional scaling (MDS) of the Atchison distance metric constructed between CIPO mice after hFMT or aFMT. The type of FMT drove the clustering (*P* = 0.001, PERMANOVA). (D) Alpha diversity, measured as the Shannon diversity index, in CIPO mice hFMT or aFMT. *P*-values were calculated using Mann–Whitney U test. (E) Taxonomic composition at the genus level in CIPO mice after hFMT and aFMT. The data are shown as relative abundance. *marks significant differences between aFMT and hFMT. *P*-values were calculated with a multivariate regression model using MaAsLin2 67, and a *P*-value < 0.05 after false discovery rate (FDR) (Q-value) correction using Benjamini–Hochberg procedure was considered significant. (F) Alpha-linolenic acid levels were assessed in cecal samples from CIPO mice after hFMT and aFMT. The data were analyzed with an unpaired Mann‒Whitney U test. A *P*-value < 0.05 was considered statistically significant.

The patient’s microbiota was consistent with the data obtained from the rest of the CIPO cohort, which was dominated by Proteobacteria, mainly Enterobacteriaceae, with a minimum proportion of Bacteroidetes and depletion of Firmicutes (Figure 1C). Only two bacterial genera differed, with *Raoultella* spp. and *Lactococcus* spp. were greater than in other CIPO patients (*Q* = 5.74*10^−6^ and *Q* = 0.011, respectively). The inferred functional profile of the patient was substantially similar to that of the rest of the CIPO cohort (data not shown), besides for an increase in polyketide sugar unit biosynthesis pathway (*Q* = 0.024), and streptomycin biosynthesis pathway (*Q* = 0.027).

### **Microbiota in humanized mice**

Germ-free mice were colonized with microbiota from the index case patient (see above) or from one healthy donor. The microbiota of the healthy donor was dominated by Firmicutes genera, such as *Ruminococcus*, *Blautia*, *Dorea*, *Clostridium* species, as well as many Lachnospiraceae species (Figure 2C), which is consistent with previously reported profiles of healthy North Americans.^36^^,^^37^

Despite some minor differences in microbiota composition between mouse recipients and human donors, similar to previous reports,^32^ a CIPO microbiota and HC profiles were transferred into gnotobiotic mice (Figure 2B, C). The microbial profiles clustered separately on principal component analysis (Permanova, *P* = 9.5*10^−5^) and were closer to their respective donors (Figure 2A). Alpha diversity was lower in mice with CIPO microbiota (Figure 2B), with CIPO mice exhibiting lower relative proportions of *Bacteroides* spp., *Parabacteroides* spp., *Alistipes* spp., *Akkermansia* spp., *Parasutterella* spp., *Collinsella* spp., and *Hungatella* spp., among others, and higher proportions of Enterobacteriaceae spp. such as *Escherichia‒Shigella*, *Klebsiella*, *Citrobacter,* and *Roultella* (Figure 2C, Supplementary Table 3).

Inferred functional profiles of the microbial communities of CIPO and HC mice revealed that, similar to the patient cohort, the CIPO microbiome was enriched in genes associated with oxidative stress (glutathione metabolism, ascorbate and aldarate metabolism, and cytochrome P450 activity, among others), alpha-linolenic acid metabolism, fluorobenzoate, benzoate and ethylbenzene degradation, fatty acids degradation, and tryptophan metabolism. In addition, pathways related to protein digestion and absorption and biotin metabolism were decreased (Figure 2D). We also found a trend toward greater alpha-linolenic acid in the stool samples of CIPO mice than in comparison to HC mice (2.28 ± 0.04 vs. 2.07 ± 0.24 ng/g feces); however, this difference did not reach statistical significance. When investigating the methane metabolism pathways (KEGG Ortholog 0068), we found that the CIPO microbiome also had a lower relative abundance of three related enzymes: heterodisulfide reductase (EC 1.8.98.1), trimethylamine methyltransferase (EC 2.1.1.250), and CO dehydrogenase (EC 1.2.7.4).

### **Functional assessment in mice with human microbiota**

An abdominal CT scan revealed a larger cecal size and a higher diameter of the small intestine in mice with CIPO microbiota (Figure 2E,F), but the size of the stomach was similar to that in mice with HC microbiota. CIPO mice exhibited markedly slower gastrointestinal transit compared to HC mice (Figure 2F). Intestinal transit was so slow in CIPO mice that most metallic beads stayed in the proximal small bowel or even stomach, while in HC mice most of the beads were located in the cecum or colon. Thus, the transfer of CIPO microbiota into mice resulted in a functional phenotype reminiscent of the CIPO patient.

CIPO mice presented altered colonic expression of genes previously associated with gastrointestinal dysmotility, such as Vasoactive Intestinal Peptide (*Vip*),^9^ piezo-1 ion channel protein (*Piezo1*),^38^ lysophosphatidic acid receptor 1 *(Lpar1*),^39^ Protease activated receptor 2 (*F2rl1*),^40^ Hypoxanthine-guanine phosphoribosyltransferase (*Hprt*)^41^, high mobility group box protein 1 (*Hmg1*),^42^ and Substance *P* (*Subp*)^43^ (Figure 2G). In addition, genes related to immune activation, such as *Nfkb*, *Myd88,* were increased in CIPO mice, while *Nos2* and *Il17A* were downregulated compared with HC mice, with multiple genes involved in barrier function being altered (*Cldn2, Ocln, Cdh1*), altogether suggesting profound changes within the neuroimmune system of the murine host.

### **FMT normalizes gastrointestinal transit, bowel size and altered microbiota profiles in mice colonized with CIPO microbiota**

Heterologous FMT with HC microbiota (hFTM) accelerated gastrointestinal transit in mice colonized with CIPO microbiota (Figure 3A) and tended to reduce cecum volume (Figure 3B,C), although it did not reach statistical significance, while autologus FMT (aFMT) had no effect.

**Figure 4.**
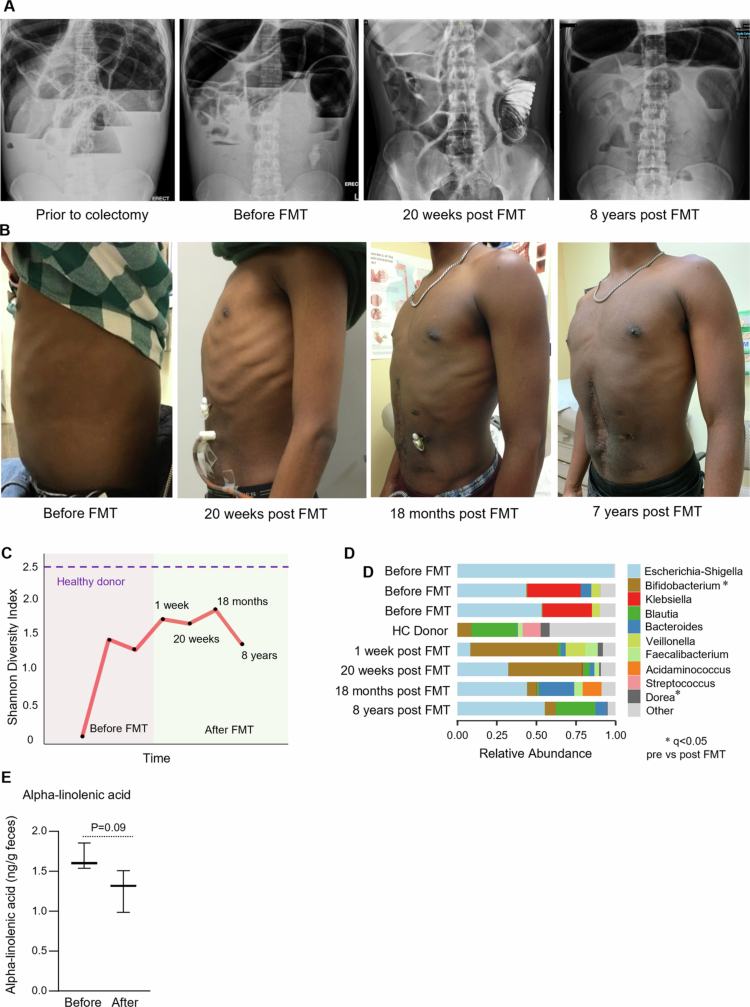
FMT induces normalization of microbiota and longterm clinical improvement in the index CIPO patient. (A) Representative abdominal X-ray images of the index CIPO patient before colectomy, before FMT, and 20 weeks and 8 y post FMT. (B) Representative photographs of the index CIPO patient before FMT, and 20 weeks, 18 months and 7 y post FMT. (C) Longitudinal representation of the alpha diversity, measured as Shannon diversity index, of the microbiome of the index CIPO patient before and after FMT. (D) Taxonomic composition at genus level of the microbiome of the index CIPO patient before and after FMT. The data is shown as relative abundance. *marks significant differences between all pooled samples before FMT and all pooled samples after FMT. *P*-values were calculated with a multivariate regression model using MaAsLin2 67 and a *P-*value < 0.05 after False Discovery Rate (FDR) correction using Benjamini–Hochberg procedure was considered significant. (E) Alpha-linolenic acid was assessed in fecal samples of the index CIPO patient before and after FMT. The data were analyzed with unpaired Mann–Whitney U test. A *P-*value < 0.05 was considered statistically significant.

No significant changes were observed in mice that received aFMT, while hFMT altered the microbiota composition of CIPO mice (Permanova, *P* = 4*10^−4^ for all groups, *P* = 0.03 for aFMT vs hFMT) (Figure 3D–F), decreasing the relative abundance of *Turicibacter* spp. and increasing the relative abundance of *Ruminococcus torques* group spp., *Alistipes*, and *Paludicola* spp. No statistically significant differences were observed in inferred microbial function (data not shown); however, FMT significantly decreased the levels of alpha-linolenic acid in the stool samples of CIPO mice (Figure 3G). These results prompted the use of FMT in the index patient.

### **FMT improves symptoms, bowel size and alters microbiota profiles in CIPO patient**

FMT from an HC was administered through a jejunostomy feeding tube on multiple occasions (for details, see Methods). The patient reported a substantial improvement in abdominal distension and discomfort within 72 h after starting FMT application (Figure 4). Three weeks later, the abdominal radiographs showed marked decrease in bowel distension and his transumbilical abdominal girth decreased from 118  cm to 80  cm, remaining low at 20 weeks (Figure 4A,B). The SF-36 Health Survey,^28^ which was administered at baseline, 3 and 20 weeks of FMT, demonstrated marked amelioration in social and physical functioning. Interestingly, the patient had high scores for both anxiety and depression that did not improve after 20 weeks of FMT (Table 3). The stool frequency increased, but the stool consistency remained unchanged. The patients’ clinical condition improved after the discontinuation of FMT at 6 months. He experienced transient worsening of gastrointestinal symptoms one month later after receiving a course of antibiotics for pneumonia, but the administration of an additional dose of FMT improved his symptoms.

Owing to his overall clinical improvement, the gastric venting tube was removed 6 months after the discontinuation of FMT. His jejunal feeding tube was removed 1 year later, and the patient was maintained on a normal diet. The patient remained basically asymptomatic for the following 6 years while initially using a probiotic mixture intermittently (VSL#3) for the first year and prucalopride 1–2 mg daily periodically on a needed basis. His body weight gradually increased, and his physical functioning improved, allowing him to actively engage in sports for the first time.

Microbial diversity increased at 1 week post-FMT (Figure 4C) and remained high for three years (Figure 4C). Indeed, the patient fecal microbiome after FMT resembled more that of the HC donor than his own microbiome prior to FMT (Figure 4D). Taxonomic analysis revealed a significant increase in bacterial genera that were abundant in the healthy donor, such as *Faecalibacterium* spp., and a significant decrease in *Enterococcus* spp. (Figure 4D). Although we observed a decrease in Enterobacteriaceae relative abundance, statistical significance was not reached after correcting for false discoveries.

**Figure 5.**
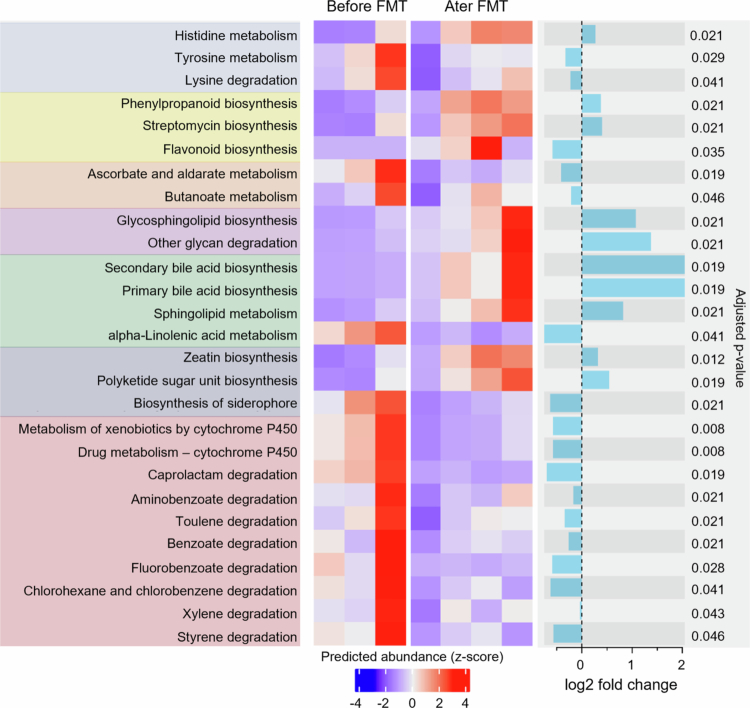
FMT alters microbial metabolic function in the index CIPO patient. Microbial metabolic pathways that differed between all pooled samples from the index CIPO patient before FMT and all pooled samples after FMT. The functional potential was predicted on the basis of marker gene sequencing profiles with PICRUSt2.^16^ Differential abundance analysis was performed using LinDA^18^ within ggpicrust2.^19^ The heatmap displays individual data for all the differentially abundant pathways. The color intensity represents z-scores, calculated from the relative abundance of each pathway. *P*-values were adjusted for FDR using the Benjamini–Hochberg procedure. A *P*-value < 0.05 after correction was considered significant.

PICRUSt2 analysis revealed a decrease in genes related to alpha-linolenic acid metabolism, which was accompanied by a numerical decrease in the level of alpha-linolenic acid in the stool after FMT (Figure 4E). In addition, we observed a decrease in the expression of genes related to fluorobenzoate and ethylbenzene degradation, likely reflecting the observed increase in strict anaerobes and Gram+ bacteria, such as Firmicutes species (Figure 5). Indeed, ethylbenzene degradation by bacteria can result in gas production, specifically in the form of carbon dioxide (CO_2_) and/or methane (CH_4_), depending on the specific metabolic pathways involved;^44^^,^^45^ thus, a reduction in ethylbenzene degradation might contribute to the decrease in intestinal gas and reduced distension observed after FMT.

## Discussion

There has been speculation regarding the role of the microbiota in the pathophysiology and disease expression of CIPO. One pilot study suggested partial symptomatic improvement in CIPO patients after FMT.^13^ Circumstantial evidence, including the exacerbation of CIPO by enteric infection and the potential benefit of rifaximin have been recently reviewed.^14^ Our work extends the field by demonstrating the microbiome profiles in 2 cohorts of CIPO patients and by providing pre-clinical and clinical evidence supporting a role for the microbiota in disease expression.

We characterized the microbiome profile of two CIPO cohorts, revealing similarities across the Canadian and Italian patients, including the dominance of the Proteobacteria phylum and depletion of Firmicutes. Colectomy, a common procedure in CIPO patients, decreases the relative abundance of several strict anaerobes, but the microbiome profile of CIPO patients remains similar to that of non-colectomized patients. The microbiota from our index patient was similar to the CIPO cohort and induced a CIPO-like phenotype when transferred into germ-free mice. This phenotype in mice was rescued by FMT from a healthy donor, thus prompting the administration of FMT to our CIPO patient, who subsequently experienced clinical improvement that was sustained over 8 y, despite the discontinuation of FMT. Our data thus provide the first profiling of the microbiome in adult CIPO patients and, importantly, provide evidence for a role of the microbiota in the pathophysiology and expression of this debilitating disorder.

In the CIPO cohort, we detected an increase in Enterobacteriaceae at the expenses of strict anaerobic bacteria from the phyla Firmicutes and Bacteroidetes, in spite of the different geographical location and different dietary habits of the two patient cohorts. Notably, most CIPO patients in both groups followed a modified diet, liquid diet, or total parenteral nutrition, which may have masked potential geographical differences. While a loss of strict anaerobes from the Firmicutes or the Bacteroidetes phyla appears to be a consistent finding, we were unable to identify a CIPO-specific microbiome signature. A recent study reported a lower Bacteroidetes relative abundance, but greater Firmicutes relative abundance, in patients with slow transit chronic constipation,^46^ while another study in women with severe slow transit constipation showed a reduction in the proportion of Firmicutes, but no dominance of Enterobacteriaceae.^47^ However, in line with our results, two other studies reported an increase in Enterobacteriaceae species, in particular *Escherichia coli* spp. in constipation-predominant irritable bowel syndrome (IBS),^48^ and a Shigella species, shown previously to inhibit peristaltic contraction, in patients with intractable functional constipation.^49^ The inhibitory effect of this bacterium was suggested to be mediated by the production of docosapentaenoic acid, an *ω*-3 fatty acid.^49^ Interestingly, inferred functional pathways of the CIPO cohort microbiome were enriched in fatty acids metabolism, such as alpha-linoleic acid and arachidonic acid, both precursors of docosapentaenoic acid, at the expenses of the metabolism of ether lipids, essential signaling molecules. Confirmatory assays revealed that alpha-linolenic acid levels were elevated in stool samples from CIPO patients. An increase in alpha-linolenic acid may not only contribute to increased levels of docosapentaenoic acid,^49^ but also reduce the production of other *ω*-3 PUFAs, such as eicosapentaenoic acid (EPA) and docosahexaenoic acid (DHA), which play critical roles in host physiology.^50^ Similarly, CIPO patients have been previously reported to have reduced expression of glial lysophosphatidic acid receptor 1 (LPAR1) in the colon and ileum, likely contributing to the severe dysmotility.^39^ Indeed, LPA, the ligand for LPAR1, which may also be a product of ether lipids metabolism (significantly reduced in our CIPO patients cohorts), was reported to reduce colonic contractility and thus modulate colonic motor complexes, likely through its proinflammatory effect on enteric glia.^39^

The decision to use the index patient in the proof-of-concept experiment was initially driven by his very poor quality of life and lack of other therapeutic options. The patient had undergone colectomy and this has been suggested to promote the bloom of Proteobacteria species, including Enterobacteriaceae, albeit to a lesser degree than observed in our patient.^51^ As CIPO may involve the proximal GI tract, colectomy may not necessarily reduce symptoms such as abdominal pain and distension, requiring additional therapy, as in the case of the index patient. We thus consider our patient to represent a prototype of CIPO patients who are persistently symptomatic despite colectomy, a group that is in need of novel therapeutic approaches.^52^

The microbiome of our index CIPO patient, although similar to that of the patient cohort, was extremely altered, consisting almost entirely of Enterobacteriaceae. Several factors may explain this kind of dysbiosis in CIPO. Delayed gastrointestinal transit *per se* has been associated with an increased prevalence of Proteobacteria.^53^^,^^54^ In addition, the low abundance of Firmicutes may contribute to the phenotype, as this phylum has been associated with faster GI transit.^47^ Additionally, as previously mentioned, colectomy may have favored increased Enterobacteriaceae.^51^ However, our comparative analysis of patients with or without colectomy suggests that colectomy decreases the relative abundance of several strict anaerobic fermentative bacteria but does not impact the relative abundance of Proteobacteria, thus suggesting that an increase in Enterobacteriaceae, as observed in our index patient, is likely more related to their severely altered gastrointestinal transit. Interestingly, antibiotic treatment has been proposed to contribute to blooms of Enterobacteriaceae as a result of a change in intestinal epithelial cells metabolism towards anaerobic glycolysis.^55^ Indeed, our index patient underwent multiple cycles of antibiotics during his illness, especially in early childhood.

We adopted a reverse-translational approach to determine the ability of the microbiota to reproduce the phenotype of altered gut function in germ-free mice, using our previously described protocol.^32^ We employed a previously validated *in vivo* technique for the measurement of GI transit using radio-opaque markers and videofluoroscopy.^33^ Colonization of germ-free mice with the index patient’s microbiota resulted in slow transit. Furthermore, an abdominal CT scan demonstrated that CIPO mice had a larger cecum and enlarged small bowel loops than mice colonized with microbiota from a healthy control. Germ-free mice are known to have cecal enlargement and slow intestinal transit that are normalized upon the introduction of bacteria.^56^^,^^57^ However, the introduction of CIPO microbiota induced slow transit and a large cecal size that likely underlies bloating in CIPO. We were unable to explain the unique mechanism by which the CIPO microbiota slows intestinal transit and increases bowel distension, but we identified multiple altered pathways, either in host neuroimmune-related genes or microbial metabolism, although we did not observe clear differences in methane-producing bacteria.^11^

Inferred metagenomics provided potential mechanisms by which the CIPO microbiota may contribute to the pathophysiology of this condition. Genes involved in amino acids and fatty acids metabolism and degradation, as well as genes related to benzoate/fluorobenzoate degradation and glutathione metabolism were upregulated in CIPO mice, similar to what we observed in CIPO patients. While most of these pathways can be traced back to the observed increase in Enterobacteriaceae and their pathogenic potential, an increase in tryptophan metabolism might potentially leading to decreased serotonin, a major neurotransmitter involved in gut motility.^58^ In addition, CIPO mice presented increased expression of microbial genes related to alpha‒linolenic acid metabolism, and a tendency for higher stool alpha‒linolenic acid, as observed in CIPO patients. On the other hand, CIPO mice presented downregulation in microbial genes related to protein digestion and absorption, which was likely associated with a loss in proteolytic bacteria. The altered expression of immunoregulatory genes, including *Myd88*, *Nfkb, and Il17a,* together with multiple genes related to intestinal barrier function (*Cldn2, Ocln, and Cdh1)* in the CIPO mouse colon raises the possibility that the CIPO microbiota activates innate immunity, leading to altered motility, as has been described in slow transit constipation.^59^^,^^60^ Our observation of increased claudin-2 and occludin expression may initially seem unexpected. However, similar changes have been reported following the loss of sodium‒potassium‒chloride cotransporter-1 (NKCC1) function in both *in vitro* and *in vivo* models.^61^ NKCC1, encoded by *SLC12A2*, is a key basolateral cotransporter, and patients with *SLC12A2* mutations present with severe gastrointestinal dysfunction, including bowel obstruction and constipation.^61^ Thus, it is plausible to speculate that in our humanized mice, an upregulation of claudin-2, occludin and e-cadherin might reflect a general physiological colonic dysfunction. Further, a lower expression of colonic piezo-1 in CIPO mice is in line with recent studies implicating piezo-1 in cholinergic neurons as a modulator of colonic motility.^38^ Finally, a lower expression of VIP in the colon of CIPO mice, reflects changes in VIP that have been reported in CIPO patients.^9^ Taken together, these findings provide a basis for further investigating the mechanisms underlying microbiota-mediated changes in motility in the context of CIPO.

The functional importance of the gut microbiota in CIPO is supported by the results of the humanized mouse model and reinforced clinically by the surprisingly rapid and long-lasting clinical improvement of our index patient after serial FMTs. As there is no established protocol for FMT in severe CIPO, especially in individuals with such extreme dysbiosis, we administered stool from the healthy donor at various frequencies, starting with daily administration and then decreasing its frequency, guided by the patient’s symptoms. We administered the FMT slurry via the jejunostomy feeding tube, which also provided easy access for self administration. The patient tolerated the treatment well, experiencing only fatigue and transient diarrhea between weeks 1 and 2, but without fever or other signs of toxicity. At that time, a decision was made to reduce the frequency of stool administration, and there was no clinical deterioration. The observed clinical improvement correlated with a significant increase in bacterial diversity, as well as an increased abundance of *Faecalibacterium* spp. that have well-described anti-inflammatory properties and are indicative of a healthy microbiota.^62^ The decrease in Enterobacteriaceae abundance and predicted reduction in alpha-linolenic acid metabolism, fluorobenzoate and ethylbenzene degradation after FMT might likely contribute to the observed clinical improvement as a result of a decrease in gas production.^44^^,^^45^ Similar findings were reported in patients with recurrent *Clostridium difficile* infection and linked to the severity of inflammation in Crohn’s disease patients.^63^^,^^64^ The observed decrease in stool alpha-linolenic acid with FMT in both CIPO mice and our index patient (*P* = 0.09) points to the importance of microbial metabolism of fatty acids, warranting further investigation.

To date, there is only one other report of FMT in a case series of nine patients with CIPO (13). FMT for six consecutive days administered via nasojejunal tube significantly relieved, but did not abolish symptoms of abdominal pain, particularly at 8 weeks follow-up, which enabled the patients to better tolerate enteral nutrition. However, the intestinal microbiota of the patients was not characterized in that study.^13^ This contrasts with our patient, who was able to eliminate the need for enteric feeding altogether and to rely fully on a normal diet, with a corresponding increase in microbial diversity.

Our study has several limitations. First, the CIPO cohort was rather small, reflecting the rarity of this patient population. Second, the mechanistic analyses were performed using the microbiota from our index patient, which may limit the generalizability of our findings. Finally, our FMT protocol was very intense, as we tried to correct the severe dysbiosis observed in the index patient. Nonetheless, our data provide valuable insights into CIPO pathophysiology and lay the groundwork for future studies in larger and more diverse patient cohorts, which could also explore other, less intense FMT regimes and test them for both safety and efficacy.

In conclusion, our study is the first detailed description of the microbiota in adult CIPO patients and provides proof of concept that the microbiota plays an important role in the clinical expression of this condition, rationalizing the use of microbial-directed therapies, including serial FMTs, to achieve marked and sustained symptomatic improvement.

## Disclosure of potential conflicts of interest

No potential conflicts of interest were disclosed.

## Supplementary Material

Supplementary material final.docx Supplementary material final.docx

## Data Availability

The main and supplementary data that support the findings of this study are available in figshare at: https://figshare.com/s/8e89fc22b20cc2153ec0. 16S rRNA gene sequencing data generated in this study have been deposited in the National Center for Biotechnology Information (NCBI), Sequence Read Archive (SRA) under accession code PRJNA1223481.
