# Supplementary material for: The role of gut microbiota in chronic intestinal pseudo-obstruction: exploring fecal microbiota transplantation as a treatment option
Source: Gut Microbes. 2026 Jan 7;18(1):2610597. doi: 10.1080/19490976.2025.2610597 (PMC12785189; doi:10.1080/19490976.2025.2610597)
Supplement: Supplementary material final.docx [file KGMI_A_2610597_SM6416.docx]

**Supplementary table 1. Different taxa (at genus level) between CIPO patients that underwent a colectomy or not.**

**Supplementary table 2. Significantly different taxa (at genus level) between CIPO colonized mice and healthy colonized mice.**

**Supplementary Figures**

**
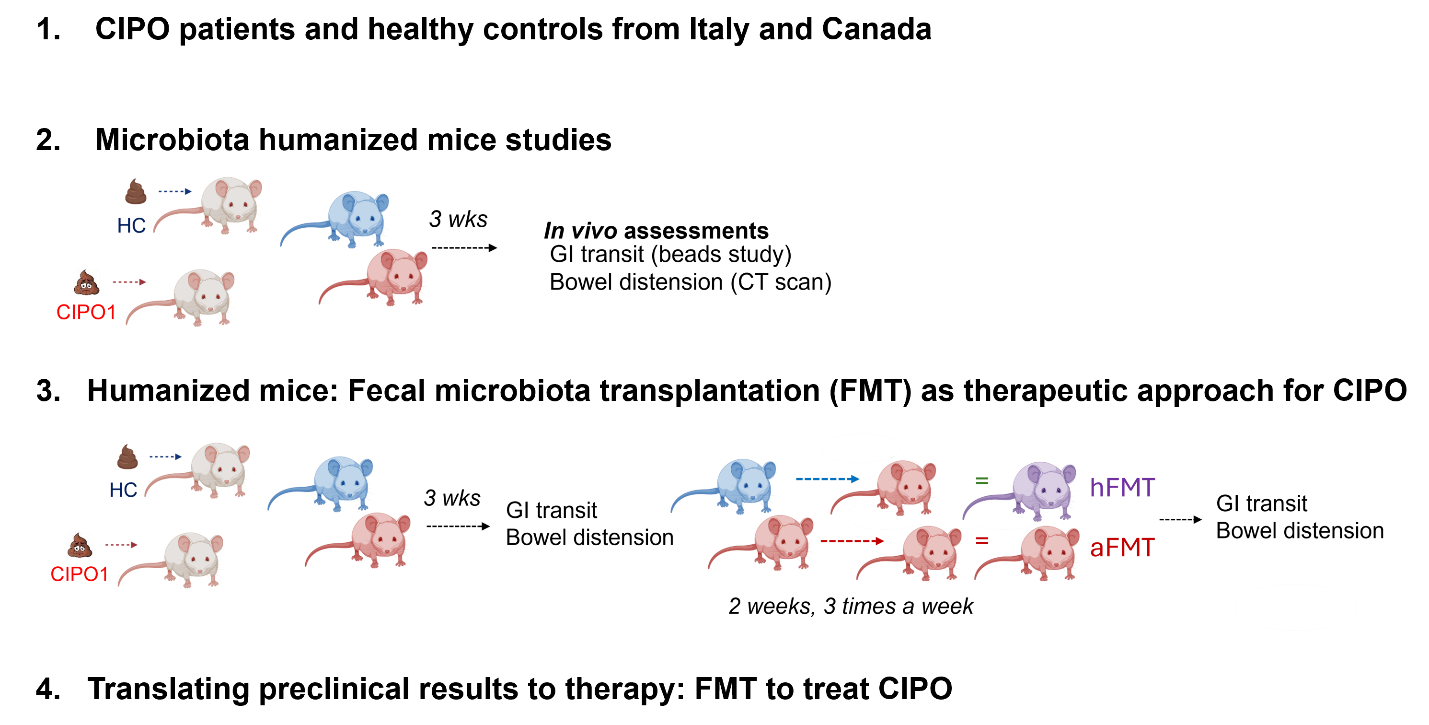
**

**Supplementary Figure 1. Experimental flow chart.**

**
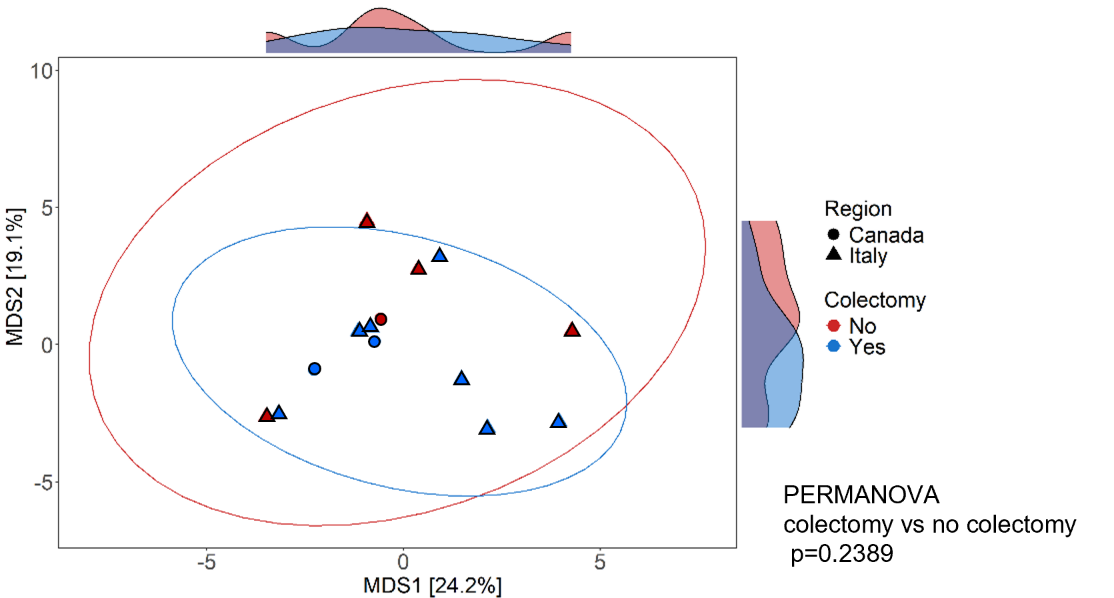
**

**Supplementary Figure 2. The microbiome of CIPO patients with and without colectomy.** Multidimensional scaling (MDS) plot of the Atchison distance metric constructed between CIPO patients that underwent partial or total colectomy (n=9) and those without colectomy (n=4) from both Canada and Italy. The clustering was not significant (P=0.24, PERMANOVA). The side panel density plots show the distribution of samples along each axis of the ordination plot.
